# Allele and Genotype Frequencies of Production Trait SNPs in Crossbred Heifers of the Colombian Caribbean

**DOI:** 10.3390/ani16203270

**Published:** 2026-10-17

**Authors:** Marcos Ahumada Velasco, Andrés Camilo Rodríguez Serrano, Juan David Corrales Álvarez, Jesús María Cárdenas Beltrán, José Morelos Gómez

**Affiliations:** 1 CTIE (Centro de Transferencia, Innovación y Emprendimiento of the Universidad de Cartagena), Universidad de Cartagena, Cartagena de Indias 130001, Colombia; camilo.rodriguez.serrano@gmail.com (A.C.R.S.); jcbestadistico1231@gmail.com (J.M.C.B.); jmorelosg@unicartagena.edu.co (J.M.G.); 2 Faculty of Agricultural Sciences, La Salle University, Bogotá 111321, Colombia; jdcorrales@unisalle.edu.co

**Keywords:** allele frequency, calpain, calpastatin, casein, dual-purpose cattle, genomic characterization, Hardy–Weinberg equilibrium, selection index

## Abstract

The Colombian Caribbean dual-purpose livestock system (*Bos taurus* × *Bos indicus*) is an important source of milk and beef, yet molecular characterization of trait-associated markers remains limited. This study characterized allele and genotype frequencies of 29 single-nucleotide polymorphisms (SNPs) associated with meat quality and milk production traits in 1585 crossbred heifers from five departments of the Colombian Caribbean. DNA was extracted from hair follicles and genotyped with the Axiom™ Bovine 100K Array. Markers spanned genes for muscle growth and proteolysis (*CAPN1*, *CAST*, *MSTN*), energy metabolism (*LEP*, *DGAT1*), and milk protein composition (*CSN2*, *CSN3*, *PAEP*, *GH1*, *GHR*, *ABCG2*). Hardy–Weinberg equilibrium (HWE) was assessed by chi-squared test. Of the 29 markers, 21 were polymorphic and testable: 19 conformed to HWE and 2 deviated (*PAEP*_239, *PAEP*_312). The remaining 8 were monomorphic. Approximately 48% of loci showed very high favorable allele frequencies (q > 0.99 or *p* > 0.99), whereas 52% maintained intermediate heterozygosity. *CSN3*_526 reached near-fixation of the favorable B allele (q = 0.996), consistent with selection for cheese yield, whereas *CAST*_282 and *CAST*_2870 showed high heterozygosity (f(AB) ≈ 0.47–0.48), indicating substantial remaining variation at these loci. These findings provide the first descriptive dual meat-and-milk SNP frequency profile for these crossbred heifers and a molecular baseline for the future development of bioeconomic selection indices in tropical dual-purpose systems. Frequencies are reported at the pooled and department-stratified levels; the results are descriptive and require phenotypic validation before any inference on marker effects or suitability for selection.

## Simple Summary

In the Caribbean region of Colombia, many small farms raise mixed-breed cattle that provide both milk and beef for rural families. Improving these animals through breeding requires knowing which inherited traits are already common in the herds and which still vary enough to be improved. In this study, we examined small differences in the DNA of 1585 young female cattle from five departments of the region and looked at genes linked to milk production, cheese-making quality, and meat tenderness. We found that some traits were already very common across almost all animals, such as a milk variant that favors cheese making, which is valuable for local farmers. Other traits related to meat tenderness still differed widely among animals, meaning that selecting the right individuals as future mothers could gradually improve meat quality without major changes to the farms. These results offer farmers and technicians a practical starting point to choose better breeding animals and to design selection programs suited to tropical conditions, helping make these family farming systems more productive and economically sustainable while caring for the animals and the environment.

## 1. Introduction

Genomic tools have become fundamental for characterizing cattle populations and identifying genetic variants associated with productive traits. Single-nucleotide polymorphisms (SNPs) distributed across the bovine genome allow efficient detection, classification, and mapping of genes related to commercially relevant phenotypes [1,2]. Commercial high-density genotyping platforms ranging from 50K to 150K markers, enable simultaneous analysis of thousands of loci and have been widely applied in European and North American breeds [3,4,5], as well as in *Bos indicus* and crossbred populations in Latin America [6,7].

For meat quality, key markers in the calpain (*CAPN1*_316, *CAPN1*_4751, *CAPN1*_530), calpastatin (*CAST*_282, *CAST*_2870, *CAST*_2959), and myostatin (*MSTN*) genes have been associated with post mortem proteolysis and tenderness [8,9,10], whereas leptin (*LEP*) polymorphisms influence energy metabolism and fat deposition [11]. For milk production and composition, polymorphisms in κ-casein (*CSN3*), β-casein (*CSN2*), β-lactoglobulin (*LGB*/*PAEP*), growth hormone (*GH1*), its receptor (*GHR*_F279Y), and diacylglycerol O-acyltransferase 1 (*DGAT1*_K232A) are associated with milk yield, protein content, fat concentration, and cheese-making properties [12,13,14,15].

In Colombia, genomic characterization has been conducted in Creole breeds such as Romosinuano [16], Hartón del Valle [17], and Blanco Orejinegro (BON) [18,19], as well as in crossbred populations [6]; however, integrated genomic information combining meat and milk trait markers in dual-purpose systems of the Colombian Caribbean remains scarce. These systems are characterized by crossbreeding *Bos taurus* × *Bos indicus* under tropical lowland conditions, generating income from simultaneous milk, cheese, and beef sales—a context in which conventional selection approaches based on single-trait indices—may be insufficient [20].

The objective of this study was to characterize allele and genotype frequencies of a panel of 29 SNPs associated with meat quality and milk production traits in crossbred heifers from dual-purpose systems across five departments of the Colombian Caribbean (Bolívar, Córdoba, La Guajira, Magdalena, and Sucre), and to assess Hardy–Weinberg equilibrium in this population to provide a molecular baseline for future bioeconomic selection indices tailored to tropical dual-purpose cattle production.

## 2. Materials and Methods

### 2.1. Study Area and Animal Sampling

The study was conducted in 25 municipalities across the departments of Bolívar, Córdoba, La Guajira, Magdalena, and Sucre, Colombia, which are representative of tropical lowland dual-purpose production systems with a mean altitude of approximately 5 m above sea level, a mean temperature of 30 °C, and an annual rainfall ranging from 500 mm (in the drier areas of La Guajira) to 2200 mm (in the more humid areas of Córdoba and Sucre).

A total of 1594 nulliparous crossbred heifers (*Bos indicus* × *Bos taurus*), aged 24–36 months, were enrolled during February 2024 (Bolívar, *n* = 122; Córdoba, *n* = 332; La Guajira, *n* = 500; Magdalena, *n* = 500; Sucre, *n* = 140). Animals were restrained in handling facilities where 30–40 tail-switch hair follicles were manually collected per individual using a rapid pull against the direction of hair growth. Samples were kept dry and individually packaged in paper envelopes to ensure traceability.

### 2.2. DNA Extraction and Genotyping

DNA was extracted from the hair follicles using a magnetic-bead protocol (MagMAX™ CORE Nucleic Acid Purification Kit, Applied Biosystems, Thermo Fisher Scientific, Austin, TX, USA). DNA concentration and purity were evaluated by spectrophotometry (NanoDrop™ spectrophotometer, Thermo Fisher Scientific, Waltham, MA, USA), accepting concentrations of 75–312 ng/µL with an A260/280 ratio between 1.8 and 2.0 as criteria for adequate quality. Genotyping was performed using the Axiom™ Bovine Genotyping 100K Array (~100,000 SNPs) on a GeneTitan™ MC Instrument (Thermo Fisher Scientific, Waltham, MA, USA) [21].

From the complete genotyped dataset, a panel of 29 candidate SNPs was selected based on previously reported associations with meat quality and milk production traits (Table 1).

### 2.3. Quality Control and SNP Filtering

Quality control was applied to ensure data reliability. SNPs and individual samples with a genotyping call rate below 90% were excluded. Hardy–Weinberg equilibrium (HWE) was evaluated for each marker by chi-squared test (χ^2^) at a significance level of *p* > 0.001 to identify SNPs with possible genotyping errors or biologically meaningful deviations. Quality control for near-fixed loci followed a configuration-based rule rather than an arbitrary numeric threshold: at any locus where the rare allele appeared exclusively as a single rare-allele homozygote in the complete absence of heterozygotes across all 1585 animals, that isolated homozygous call was flagged as biologically implausible under Mendelian expectation, because a rare homozygote cannot arise without heterozygous carriers, and was therefore treated as a probable genotyping or clustering artifact. Each flagged call was verified by re-inspecting the raw allele-intensity cluster plots for the corresponding Axiom assay, where it fell outside the main genotype cluster. In the present dataset, this configuration occurred at a single locus and involved a single genotype call: at *MSTN*_C313Y, one animal carried an isolated rare-allele homozygote (1 rare homozygote vs. 1584 common homozygotes and no heterozygotes). This call was recoded to the common homozygote, rendering the locus monomorphic, and all frequency and HWE analyses were re-run to confirm that the correction did not alter the qualitative conclusions.

### 2.4. SNP Panel and Favorable Allele Definition

The 29 SNPs were grouped by biological function: (i) meat quality and muscular development—calpain (*CAPN1*_316, *CAPN1*_4751, *CAPN1*_530), calpastatin (*CAST*_282, *CAST*_2870, *CAST*_2959), and myostatin (*MSTN*_C313Y, *MSTN*_E226X, *MSTN*_E291X, *MSTN*_Q204X, *MSTN*_F94L, *MSTN*_S105C); (ii) energy metabolism and fat deposition—leptin (*LEP*_252T, *LEP*_59V, *LEP*_A963); and (iii) milk production and composition—β-casein (*CSN2*_245, *CSN2*_411), κ-casein (*CSN3*_470, *CSN3*_526), β-lactoglobulin/*PAEP* (*PAEP*_181, *PAEP*_225, *PAEP*_237, *PAEP*_239, *PAEP*_312, *LGB*_1, *LGB*_2), growth hormone (*GH1*_2141, *GH1*_2291), *GHR* (*GHR*_F279Y), and *DGAT1* (*DGAT1*_K232A).

Favorable alleles were defined based on reported functional evidence in the scientific literature and encoded under an additive model (0 = homozygous unfavorable, 1 = heterozygous, 2 = homozygous favorable). For *MSTN*, the wild-type (non-mutated) allele was considered favorable due to its association with better reproductive performance and lower dystocia incidence (Table 1).

### 2.5. Statistical Analysis

For each SNP, observed genotype frequencies (AA, AB, BB) were estimated from the filtered sample (*n* = 1585). Allele frequencies were calculated as follows:

*p = fA = (*2*NAA + NAB)/*2*N and q = fB = (*2*NBB + NAB)/*2*N*

where *NAA*, *NAB*, and *NBB* are the numbers of individuals with genotypes AA, AB, and BB, respectively, and *N* = *NAA* + *NAB* + *NBB* is the total number of individuals; *p* (*fA*) and *q* (*fB*) denote the frequencies of alleles *A* and *B*.

Observed heterozygosity (Ho) and expected heterozygosity (He), reported here in full at first use, were computed as Ho = NAB/N and He = 2pq. Expected HWE genotype frequencies were compared to observed frequencies by chi-squared test (χ^2^, 1 degree of freedom, χ^2^crit(0.05;1) = 3.84; α = 0.05). All analyses were performed in IBM SPSS Statistics v25 (IBM Corp., Armonk, NY, USA).

## 3. Results

### 3.1. DNA Quality and Sample Retention

DNA concentration across the 1594 samples ranged from 75 to 312 ng/µL (mean ≈ 185 ng/µL), with A260/280 ratios within acceptable limits (1.8–2.0), confirming adequate quality for high-density array genotyping. After quality control (call rate ≥ 90%), 1585 samples (99.4%) were retained for analysis; nine samples failed the quality threshold and were excluded.

### 3.2. Hardy–Weinberg Equilibrium

Of the 29 candidate SNPs, 21 were polymorphic and testable for Hardy–Weinberg equilibrium; of these, 19 conformed (*p* > 0.05) and two deviated significantly: *PAEP*_239 (χ^2^ = 4.08; *p* = 0.044) and *PAEP*_312 (χ^2^ = 6.03; *p* = 0.014). The remaining 8 markers were monomorphic, so the test was not applicable. During quality control, a single isolated rare-allele homozygote (AA) at *MSTN*_C313Y—observed without any heterozygotes and inconsistent with Mendelian expectation at a near-fixed locus—was identified as a probable genotyping error and recoded accordingly; *MSTN*_C313Y was therefore treated as monomorphic. Per-locus χ^2^ statistics and *p*-values are reported in Table 2. For loci with very high allele frequencies (*p* ≥ 0.95 or q ≥ 0.95), interpretation of the chi-squared test requires caution given the reduced statistical power of the test under conditions of low genetic variability.

### 3.3. Allele and Genotype Frequencies

Allele frequencies (A and B) for the full panel of 29 SNPs are shown in Figure 1, and the corresponding observed genotype frequencies (AA, AB, and BB) are shown in Figure 2.

#### 3.3.1. Meat Quality Marker Frequencies

Within the calpain system, *CAPN1*_316 showed a high frequency of the AA genotype (f(AA) = 0.929; allele A: *p* = 0.964), with complete absence of the BB genotype, indicating near-fixation of the A allele. Notably, the A allele does not correspond to the favorable allele for tenderness (the favorable C allele of Table 1 confers improved tenderness; throughout, the generic codes AA, AB and BB denote the favorable homozygote, heterozygote and alternative homozygote defined per locus in Table 1, so for *CAPN1*_316 AA = CC, AB = CG, BB = GG); therefore, its near-fixation represents a constraint for genetic improvement of this trait. *CAPN1*_530 displayed a high frequency of the BB genotype (f(BB) = 0.878; allele B: q = 0.938), indicating near-fixation in the opposite direction. In contrast, *CAPN1*_4751 exhibited greater genetic variability, with a substantial proportion of heterozygotes (f(AB) = 0.263) (Table 2).

Among the calpastatin loci, *CAST*_282 and *CAST*_2870 exhibited the highest heterozygosity in the panel, with f(AB) = 0.478 and 0.474, respectively, and balanced allele frequencies (*CAST*_282: A = 0.533, B = 0.467; *CAST*_2870: A = 0.565, B = 0.435). *CAST*_2959 also presented intermediate heterozygosity (f(AB) = 0.437; A = 0.644, B = 0.356) and conformed to HWE (χ^2^ = 3.45; *p* = 0.063). All six *MSTN* markers evaluated (*MSTN*_C313Y, *MSTN*_E226X, *MSTN*_E291X, *MSTN*_F94L, *MSTN*_Q204X, *MSTN*_S105C) showed complete fixation of the wild-type allele (q = 1.000), as reflected by the exclusive presence of the BB genotype in the population. At *MSTN*_C313Y, a single isolated AA genotype—observed without any heterozygotes—was identified as a probable genotyping error during quality control and recoded; the locus was consequently treated as monomorphic (Table 2).

#### 3.3.2. Milk Production Marker Frequencies

*CSN3*_526 showed a very high frequency of the favorable B allele (q = 0.996; f(BB) = 0.992; minor-allele count MAC = 14), which has been associated with improved milk coagulation properties and cheese yield. *CSN3*_470 showed predominantly the BB genotype (f(BB) = 0.785; B allele frequency q = 0.886). For β-casein, *CSN2*_245 exhibited a high frequency of the AA genotype (f(AA) = 0.826), corresponding to the A2A2 variant, which has been associated with differentiated value in specialty milk markets.

Among *PAEP* loci, *PAEP*_181 and *PAEP*_225 were completely fixed for the BB and AA genotypes, respectively (f = 1.000). In contrast, *PAEP*_239 showed intermediate heterozygosity (f(AB) = 0.477; A = 0.652, B = 0.348) and *PAEP*_312 also showed substantial heterozygosity (f(AB) = 0.412; A = 0.264, B = 0.736); both *PAEP* loci deviated significantly from HWE in the pooled sample (*PAEP*_239: χ^2^ = 4.08, *p* = 0.044; *PAEP*_312: χ^2^ = 6.03, *p* = 0.014); department-stratified testing (Table S3) showed that *PAEP*_239 conformed to equilibrium in all five departments and *PAEP*_312 in four of five, indicating that these pooled departures reflect population subdivision (a Wahlund effect) rather than genotyping error.

*LEP*_A252T showed a very high frequency of the B allele (f(BB) = 0.984; q = 0.992; MAC = 26), consistent with historical selection pressure related to energy efficiency. *ABCG2* showed a very high frequency of the favorable A allele (f(AA) = 0.994; *p* = 0.997; MAC = 10). *GH1*_2141 and *GHR*_F279Y also showed genotype frequencies close to fixation (f(BB) = 0.954 and f(AA) = 0.967, respectively), while *GH1*_2291 maintained more balanced allele frequencies (A = 0.811, B = 0.189; f(AB) = 0.293). *DGAT1* showed intermediate variation (A = 0.831; f(AB) = 0.284).

## 4. Discussion

This study provides the first systematic characterization of a dual meat-and-milk SNP panel in crossbred heifers from five departments of the Colombian Caribbean, encompassing 1585 individuals, a sample size substantially larger than previous Colombian genomic studies, which typically included 100–500 animals [17,18,19].

### 4.1. Meat Quality Markers

The near-fixation of the non-favorable A allele at *CAPN1*_316 (*p* = 0.964) and the predominance of the BB genotype at *CAPN1*_530 (f(BB) = 0.878) are consistent with patterns reported in *Bos indicus* populations and Latin American crossbreds, where the favorable allele for tenderness tends to be at low frequency [8,22]. Similar results have been described in Brahman and Nelore populations, where the non-favorable allele for *CAPN1* tenderness markers tends to be fixed or near-fixed [9,22]. These findings contrast with specialized taurine beef breeds (Angus, Hereford), where sustained selection pressure has raised favorable allele frequencies above 0.60 [8,23]. The intermediate heterozygosity profile of *CAPN1*_4751 (f(AB) = 0.263) and the high heterozygosity of *CAST*_282 (f(AB) = 0.479) and *CAST*_2870 (f(AB) = 0.474), with balanced allele frequencies (A ≈ 0.53–0.57), indicate segregating genetic variation for calpastatin-mediated tenderness [10,24,25]. These patterns mirror those reported in Colombian *Bos indicus*-influenced populations (heterozygote frequencies 0.30–0.50; [26,27]), attributable to crossbreeding history and historical prioritization of adaptive over meat quality traits.

The complete fixation observed at all *MSTN* loci is expected, as the loss-of-function mutations in myostatin are rare even in specialized beef breeds and carry negative pleiotropic effects on reproduction and calving ease [28,29]. Their absence in the evaluated population confirms normal muscular development pathways and rules out double-muscling in these herds.

The intermediate heterozygosity at *CAST* loci and *CAPN1*_4751 implies that a proportion of animals carry the favorable allele. Near-fixation at an individual candidate locus constrains the discriminating power of that specific marker between animals but does not indicate absence of genetic variation for tenderness, which is polygenic and governed by many loci beyond the candidate panel evaluated here; likewise, allele frequency alone describes the distribution of variants in the population and does not demonstrate the direction or magnitude of a marker’s effect on the phenotype. To illustrate the potential magnitude of response without asserting validated effects, a single-generation truncation-selection approximation (Δp ≈ i·h^2^·α·p·q) for the two variable *CAST* loci, using the observed frequencies (*CAST*_282, *p* = 0.533; *CAST*_2870, *p* = 0.565), a moderate heritability for Warner–Bratzler shear force (h^2^ = 0.30), a conservative standardized allele substitution effect (α ≈ 0.10 phenotypic SD, of the order reported for calpastatin markers on carcass and meat quality traits in *Bos indicus*-influenced and crossbred cattle [24]), and selection of the top 10% of animals (i = 1.755), yields an expected one-generation change of only Δp ≈ 0.013 for both loci. This illustrative value, presented under explicitly stated assumptions rather than as an empirical estimate, indicates that per-generation gains through these individual markers would be modest and would depend on validated marker effects, linkage phase, and the joint polygenic architecture of the trait. This many-small-effects expectation is consistent with a genome-wide association analysis of a conformation-based reproductive index in a related dual-purpose crossbred population from the same region, which revealed no major effect loci [30].

### 4.2. Milk Production and Composition Markers

The near-complete fixation of the B allele at *CSN3*_526 (q = 0.996; f(BB) = 0.992) is consistent with reports from Colombian dairy and dual-purpose systems oriented toward artisanal cheese production, where frequencies of the κ-casein B allele are typically reported above 0.80 [31,32]. Compared to European Holstein populations, where the B allele frequency is usually below 0.30 [13,14], the evaluated crossbreds present an intrinsic genetic advantage for cheese-making efficiency, a trait of direct economic relevance in the Caribbean rural economy. The very high favorable allele frequency at this locus limits selective progress through this specific marker only; other loci affecting the same trait retain substantial variation—for example, *CSN3*_470 (q = 0.885; MAC = 363)—and the high B-allele frequency represents a favorable baseline for the entire population.

The high frequency of the A2A2 genotype for *CSN2*_245 (f(AA) = 0.826) is noteworthy because A2 β-casein has gained commercial relevance in specialty milk markets [12]. Combined with the *CSN3*_526 profile, these findings suggest that the genetic makeup of the evaluated population aligns with high-value dairy product characteristics without deliberate selection for these traits.

Intermediate heterozygosity at *PAEP*_239 (f(AB) = 0.477) and *PAEP*_312 (f(AB) = 0.412) suggests exploitable genetic variation for milk protein traits in the *PAEP* (previously annotated as *LGB*/whey protein) gene cluster [33]. The significant departure from HWE at both loci in the pooled sample (*p* = 0.044 and *p* = 0.014, respectively) is most plausibly explained by population subdivision among the five sampled departments (a Wahlund effect): when HWE was tested separately within each department (Table S3), *PAEP*_239 conformed to equilibrium in all five departments and *PAEP*_312 in four of five (deviating only in Sucre, the smallest sample), indicating that the pooled departures arise from combining subpopulations with differing allele frequencies rather than from genotyping error. A principal component analysis of the 21 polymorphic SNPs (Figure S1) confirmed only mild differentiation among departments (the first two components explained 9.7% and 8.8% of the variance, with extensive overlap), and a heatmap of favorable allele frequency by department (Figure S2) showed largely consistent frequencies across regions. The near-complete fixation of *ABCG2* (AA genotype: f = 0.994), *GHR*_F279Y (AA: f = 0.967), and *LEP*_A252T (BB: f = 0.984) reflects historical selection patterns for adaptability and metabolic efficiency in tropical environments [11,15,34,35].

### 4.3. Implications for Genomic Selection

The heterogeneous distribution of genetic variability across the 29 SNP loci—with 48% near fixation and 52% maintaining intermediate variation—has direct implications for the design of selection indices. Markers with frequencies near fixation contribute limited discriminating power between individuals, whereas those with intermediate heterozygosity (*CAST*_282, *CAST*_2870, *CAST*_2959, *CAPN1*_4751, *PAEP*_239, *PAEP*_312, *GH1*_2291, *DGAT1*) can effectively differentiate animals and contribute to selection response [36].

The principal limitation of the present study is the absence of individual phenotypic records for the traits associated with the genotyped markers (meat tenderness, milk yield and composition, cheese-making properties), which precludes the estimation of SNP effects and genomic breeding values. Consequently, this work should be interpreted strictly as a descriptive molecular baseline survey of candidate marker frequencies, not as a predictive or validated selection tool: allele frequencies describe the distribution of variants in the population but cannot, on their own, establish the direction or magnitude of a marker’s effect on the phenotype. In addition, individual breed composition and genomic ancestry proportions and pedigree/genomic relatedness among animals were not characterized, so the reported frequencies are descriptive population-level estimates conditioned on the sampling scheme rather than ancestry-corrected parameters. Future work should integrate phenotypic records for tenderness, milk coagulation properties, and composition to estimate allele substitution effects and validate marker utility in this population.

## 5. Conclusions

Characterization of 29 SNPs in 1585 crossbred heifers from the Colombian Caribbean revealed a heterogeneous genetic structure, with approximately 48% of markers near fixation and 52% maintaining intermediate heterozygosity, and with 19 of the 21 polymorphic markers conforming to Hardy–Weinberg equilibrium (the remaining 8 markers were monomorphic). Department-stratified analysis showed that, of the two loci deviating from equilibrium in the pooled sample (*PAEP*_239, *PAEP*_312), *PAEP*_239 conformed to equilibrium in all departments and *PAEP*_312 in four of five, indicating that the pooled deviations largely reflect population subdivision rather than genotyping error. A very high frequency of the favorable κ-casein B allele (*CSN3*_526: q = 0.996; minor-allele count MAC = 14) provides a favorable baseline for cheese-making efficiency across the region, although limited variation at this specific marker constrains progress through it; other loci affecting the same trait, such as *CSN3*_470 (q = 0.885; MAC = 363), remain variable. Intermediate heterozygosity at *CAST*_282 and *CAST*_2870 (f(AB) = 0.478 and 0.474, respectively) identifies these loci as candidate targets whose usefulness for improving meat tenderness would require validation against individual phenotypic records. The near-complete fixation of all evaluated *MSTN* loci confirms the absence of double-muscling mutations and normal muscular development. This is the first systematic descriptive characterization of a combined meat-and-milk SNP panel in Colombian Caribbean crossbred cattle at regional scale, providing a molecular baseline for the future development of bioeconomic selection indices adapted to tropical dual-purpose production systems. Because the study reports marker frequencies without individual ancestry, relatedness, or phenotypic data, its conclusions are descriptive; estimation of allele substitution effects and validation of marker utility in this population remain necessary next steps.

## Figures and Tables

**Figure 1:**
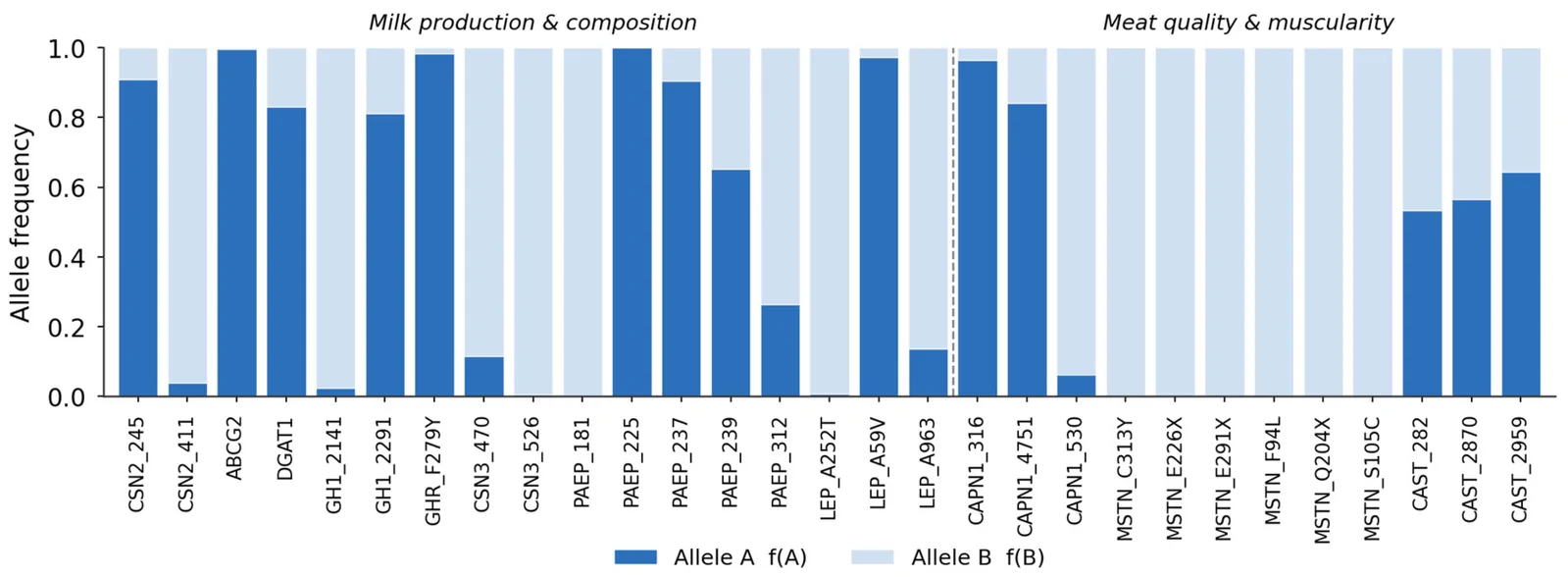
Allele frequencies (A and B) for 29 SNPs associated with meat quality and milk production traits in crossbred heifers from the Colombian Caribbean (*n* = 1585). The vertical dashed line separates SNPs associated with milk production and composition (left) from those associated with meat quality and muscularity (right).

**Figure 2:**
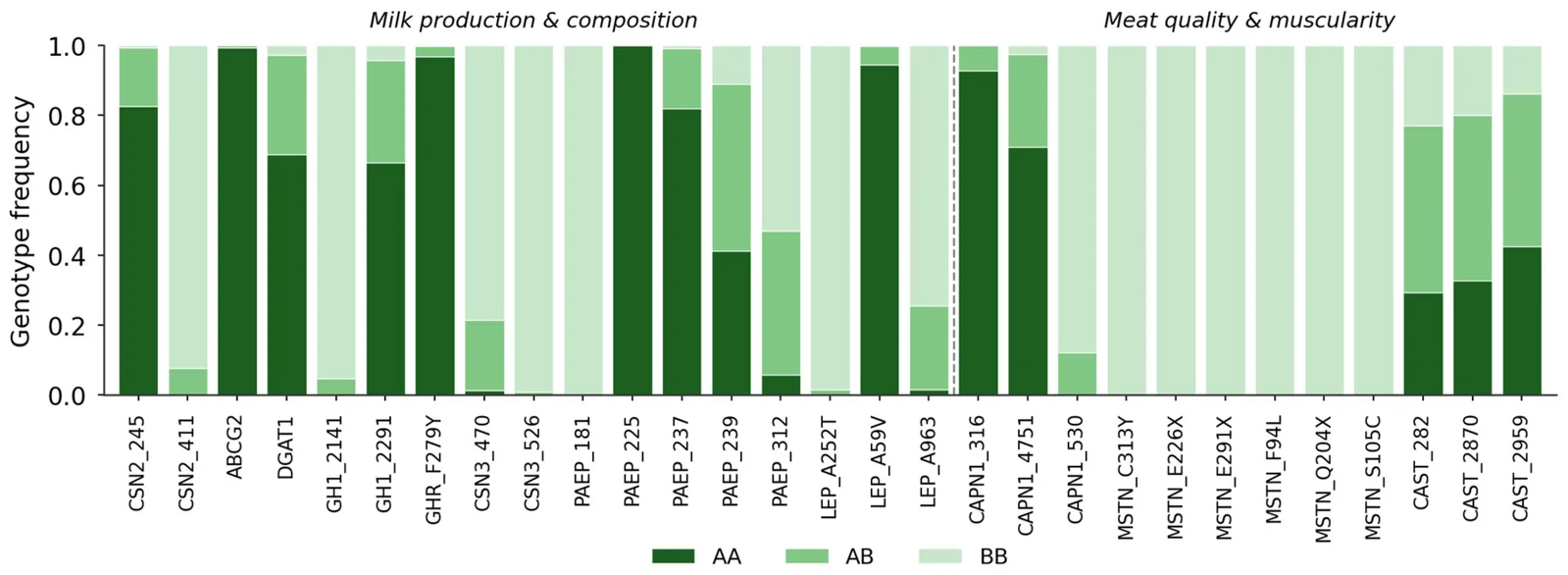
Observed genotype frequencies (AA, AB, and BB) for 29 SNPs associated with meat quality and milk production traits in crossbred heifers from the Colombian Caribbean (*n* = 1585). The vertical dashed line separates SNPs associated with milk production and composition (left) from those associated with meat quality and muscularity (right).

**Table 1:** Favorable alleles and additive coding scheme for the 29 SNPs associated with productive traits in dual-purpose cattle.

SNP	Gene	Associated Trait	Favorable Allele	Coding (Genotype = Value)
*CSN2*_245	*CSN2*	Milk protein (β-casein)	A2	A2A2 = 2, A1A2 = 1, A1A1 = 0
*CSN2*_411	*CSN2*	Milk protein (β-casein)	A2	A2A2 = 2, A1A2 = 1, A1A1 = 0
*CSN3*_470	*CSN3*	Cheese yield (κ-casein)	B	BB = 2, AB = 1, AA = 0
*CSN3*_526	*CSN3*	Cheese yield (κ-casein)	B	BB = 2, AB = 1, AA = 0
*PAEP*_181	*PAEP*	Milk protein	B	BB = 2, AB = 1, AA = 0
*PAEP*_225	*PAEP*	Milk protein	B	BB = 2, AB = 1, AA = 0
*PAEP*_237	*PAEP*	Milk protein	B	BB = 2, AB = 1, AA = 0
*PAEP*_239	*PAEP*	Milk protein	B	BB = 2, AB = 1, AA = 0
*PAEP*_312	*PAEP*	Milk protein	B	BB = 2, AB = 1, AA = 0
*DGAT1*	*DGAT1*	Milk fat content	A	AA = 2, AG = 1, GG = 0
*ABCG2*	*ABCG2*	Milk yield and solids	A	AA = 2, AB = 1, BB = 0
*GH1*_2141	*GH1*	Growth	G	GG = 2, AG = 1, AA = 0
*GH1*_2291	*GH1*	Growth	G	GG = 2, AG = 1, AA = 0
*GHR*_F279Y	*GHR*	Milk yield	Y	YY = 2, FY = 1, FF = 0
*CAPN1*_316	*CAPN1*	Meat tenderness	C	CC = 2, CG = 1, GG = 0
*CAPN1*_530	*CAPN1*	Meat tenderness	A	AA = 2, AG = 1, GG = 0
*CAPN1*_4751	*CAPN1*	Meat tenderness	C	CC = 2, TC = 1, TT = 0
*CAST*_282	*CAST*	Meat tenderness	C	CC = 2, CG = 1, GG = 0
*CAST*_2870	*CAST*	Meat tenderness	A	AA = 2, AB = 1, BB = 0
*CAST*_2959	*CAST*	Meat tenderness	A	AA = 2, AB = 1, BB = 0
*MSTN*_C313Y	*MSTN*	Muscularity	C	CC = 2, CY = 1, YY = 0
*MSTN*_E226X	*MSTN*	Muscularity	E	EE = 2, EX = 1, XX = 0
*MSTN*_E291X	*MSTN*	Muscularity	E	EE = 2, EX = 1, XX = 0
*MSTN*_Q204X	*MSTN*	Muscularity	Q	QQ = 2, QX = 1, XX = 0
*MSTN*_S105C	*MSTN*	Muscularity	S	SS = 2, SC = 1, CC = 0
*MSTN*_F94L	*MSTN*	Muscularity	F	FF = 2, FL = 1, LL = 0
*LEP*_252T	*LEP*	Energy metabolism/body fat	T	TT = 2, AT = 1, AA = 0
*LEP*_59V	*LEP*	Energy metabolism	V	VV = 2, AV = 1, AA = 0
*LEP*_963T	*LEP*	Energy metabolism	T	TT = 2, AT = 1, AA = 0

***Note.*** 0 = heterozygous, and 2 = homozygous for the favorable allele. For *MSTN*, the favorable allele corresponds to the wild-type (non-mutated) variant. Gene abbreviations: *CSN2* = β-casein; *CSN3* = κ-casein; *PAEP* = progestogen-associated endometrial protein; *DGAT1* = diacylglycerol O-acyltransferase 1; *ABCG2* = ATP-binding cassette sub-family G member 2; *GH1* = growth hormone 1; *GHR* = growth hormone receptor; *CAPN1* = calpain 1; *CAST* = calpastatin; *MSTN* = myostatin; *LEP* = leptin.

**Table 2:** Allele and genotype frequencies (observed and Hardy–Weinberg-expected), chi-squared statistic, and *p*-value for 29 SNPs in crossbred heifers from the Colombian Caribbean (*n* = 1585).

Marker	f(A)	f(B)	Obs. AA	Obs. AB	Obs. BB	Exp. AA	Exp. AB	Exp. BB	χ^2^	*p*
***Milk Production and Composition Markers***										
*CSN2*_245	0.910	0.090	0.826	0.167	0.007	0.828	0.164	0.008	0.34	0.561
*CSN2*_411	0.038	0.962	0.001	0.076	0.924	0.001	0.074	0.925	0.84	0.360
*ABCG2*	0.997	0.003	0.994	0.006	0.000	0.994	0.006	0.000	0.02	0.900
*DGAT1*	0.831	0.169	0.688	0.285	0.026	0.690	0.281	0.029	0.35	0.553
*GH1*_2141	0.024	0.976	0.001	0.047	0.952	0.001	0.047	0.952	0.00	0.945
*GH1*_2291	0.812	0.188	0.666	0.292	0.042	0.659	0.306	0.035	3.14	0.076
*GHR*_F279Y	0.984	0.016	0.968	0.032	0.001	0.967	0.032	0.000	0.80	0.372
*CSN3*_470	0.115	0.885	0.013	0.203	0.784	0.013	0.203	0.784	0.00	0.957
*CSN3*_526	0.004	0.996	0.000	0.009	0.991	0.000	0.009	0.991	0.03	0.860
*PAEP*_181	0.000	1.000	0.000	0.000	1.000	0.000	0.000	1.000	**—**	**—**
*PAEP*_225	1.000	0.000	1.000	0.000	0.000	1.000	0.000	0.000	**—**	**—**
*PAEP*_237	0.906	0.094	0.820	0.173	0.008	0.821	0.170	0.009	0.35	0.554
*PAEP*_239	0.652	0.348	0.413	0.477	0.110	0.425	0.454	0.121	4.08	0.044 *
*PAEP*_312	0.263	0.737	0.057	0.412	0.531	0.069	0.388	0.543	6.03	0.014 *
*LEP*_A252T	0.008	0.992	0.000	0.016	0.984	0.000	0.016	0.984	0.11	0.742
*LEP*_A59V	0.972	0.028	0.944	0.055	0.001	0.945	0.055	0.001	0.05	0.818
*LEP*_A963	0.136	0.864	0.016	0.240	0.744	0.019	0.235	0.746	0.54	0.463
***Meat Quality and Muscularity Markers***										
*CAPN1*_316	0.964	0.036	0.929	0.071	0.000	0.930	0.069	0.001	2.17	0.141
*CAPN1*_4751	0.842	0.158	0.710	0.264	0.026	0.709	0.266	0.025	0.09	0.767
*CAPN1*_530	0.062	0.938	0.002	0.121	0.878	0.004	0.117	0.880	1.81	0.179
*MSTN*_C313Y	0.000	1.000	0.000	0.000	1.000	0.000	0.000	1.000	—	—
*MSTN*_E226X	0.000	1.000	0.000	0.000	1.000	0.000	0.000	1.000	—	—
*MSTN*_E291X	0.000	1.000	0.000	0.000	1.000	0.000	0.000	1.000	—	—
*MSTN*_F94L	0.000	1.000	0.000	0.000	1.000	0.000	0.000	1.000	—	—
*MSTN*_Q204X	0.000	1.000	0.000	0.000	1.000	0.000	0.000	1.000	—	—
*MSTN*_S105C	0.000	1.000	0.000	0.000	1.000	0.000	0.000	1.000	—	—
*CAST*_282	0.533	0.467	0.294	0.478	0.228	0.284	0.498	0.218	2.45	0.118
*CAST*_2870	0.565	0.435	0.327	0.474	0.198	0.319	0.492	0.190	1.94	0.164
*CAST*_2959	0.644	0.356	0.425	0.437	0.138	0.415	0.459	0.127	3.45	0.063

***Note.*** Degrees of freedom were added following the reviewer’s observation; * indicates significant deviation from HWE (*p* < 0.05); “—” denotes monomorphic markers for which the test is not applicable.

## Data Availability

Individual genotype data are available as Supplementary Material (Table S1). Additional data are available upon reasonable request from the corresponding author.

## Supplementary Materials

The following supporting information can be downloaded at https://www.mdpi.com/article/10.3390/ani16203270/s1, Table S1. Individual genotype matrix for 29 SNPs in 1585 crossbred heifers. Table S2. Complete allele and genotype frequencies, including Hardy–Weinberg test statistics (χ^2^, *p*-value), for all 29 SNPs. Table S3. Allele and genotype frequencies and per-department Hardy–Weinberg tests for the 29 SNPs, stratified by department (La Guajira, Magdalena, Bolívar, Córdoba, Sucre). Figure S1. Principal component analysis of the 21 polymorphic SNPs by department. Figure S2. Heatmap of favorable allele frequency for each of the 29 SNPs across the five departments.
